# Quantitative Analyses of Schizophrenia-Associated Metabolites in Serum: Serum D-Lactate Levels Are Negatively Correlated with Gamma-Glutamylcysteine in Medicated Schizophrenia Patients

**DOI:** 10.1371/journal.pone.0101652

**Published:** 2014-07-08

**Authors:** Takeshi Fukushima, Hideaki Iizuka, Ayaka Yokota, Takehiro Suzuki, Chihiro Ohno, Yumiko Kono, Minami Nishikiori, Ayaka Seki, Hideaki Ichiba, Yoshinori Watanabe, Seiji Hongo, Mamoru Utsunomiya, Masaki Nakatani, Kiyomi Sadamoto, Takashi Yoshio

**Affiliations:** 1 Department of Analytical Chemistry, Faculty of Pharmaceutical Sciences, Toho University, Funabashi-shi, Chiba, Japan; 2 Nanko Clinic of Psychiatry, Himorogi group, Medical Corporation JISENKAI, Shirakawa-shi, Fukushima, Japan; 3 Public Interest Incorporated Foundation, Sumiyoshi-kaiseikai Sumiyoshi hospital, Koufu-shi, Yamanashi, Japan; 4 Department of Clinical Pharmacy, Yokohama College of Pharmacy, Yokohama-shi, Kanagawa, Japan; 5 Department of Clinical Pharmacy, Faculty of Pharmaceutical Sciences, Toho University, Funabashi-shi, Chiba, Japan; The Nathan Kline Institute, United States of America

## Abstract

The serum levels of several metabolites are significantly altered in schizophrenia patients. In this study, we performed a targeted analysis of 34 candidate metabolites in schizophrenia patients (*n* = 25) and compared them with those in age- and gender-matched healthy subjects (*n* = 27). Orthogonal partial least square-discriminant analysis revealed that complete separation between controls and patients was achieved based on these metabolites. We found that the levels of γ-glutamylcysteine (γ-GluCys), linoleic acid, arachidonic acid, D-serine, 3-hydroxybutyrate, glutathione (GSH), 5-hydroxytryptamine, threonine, and tyrosine were significantly lower, while D-lactate, tryptophan, kynurenine, and glutamate levels were significantly higher in schizophrenia patients compared to controls. Using receiver operating characteristics (ROC) curve analysis, the sensitivity, specificity, and the area under curve of γ-GluCys, a precursor of GSH, and D-lactate, a terminal metabolite of methylglyoxal, were 88.00%, 81.48%, and 0.8874, and 88.00%, 77.78%, and 0.8415, respectively. In addition, serum levels of D-lactate were negatively correlated with γ-GluCys levels in patients, but not in controls. The present results suggest that oxidative stress-induced damage may be involved in the pathogenesis of schizophrenia.

## Introduction

Schizophrenia is a severe psychiatric disease that affects approximately 1% of the world’s population; it is comprised of positive and negative symptoms and cognitive deficits, with an onset during adolescence [1]. Optimal treatment thus requires early detection and suitable medication, which in turn require highly trained psychiatrists. Recently, metabonomic studies have revealed significant alterations in endogenous metabolites, including amino acids, polyunsaturated fatty acids (PUFAs), myo-inositol, and citrate, in the serum of schizophrenia patients [2]–[4]. These alterations reflect the impairment of systemic metabolism in peripheral tissues, and indicate that schizophrenia should be regarded as not only a dysfunction of the central nervous system (CNS), but also as a disorder of systemic metabolism. In previous studies, however, the discriminatory power of metabonomics was found to be heavily dependent on the capacity of the instruments used for analysis; consequently, several different metabolites have been proposed as crucial factors in the pathogenesis of schizophrenia.

In the present study, we focused on metabolites that have previously been suggested to be associated with schizophrenia. For example, the glutamate hypothesis of schizophrenia etiology suggests that endogenous D-serine is a crucial factor related to the hypofunction of the *N*-methyl-D-aspartate (NMDA) receptor [5]–[7]. Oxidative stress is also associated with schizophrenia [8], and a low level of the antioxidant glutathione (GSH) has been reported in schizophrenia patients [9]. Thus, we selected 34 target metabolites, many of which have been implicated in schizophrenia, and performed quantitative analyses of their levels in serum from schizophrenia patients. Subsequently, we applied a multivariate analysis between controls and the patients, and a correlation matrix approach to determine whether there was any association between these metabolites.

## Methods

### 1. Participants

Table 1 lists the demographic features of the patients and controls that participated in this study. Twenty-five schizophrenia patients (11 men and 14 women) were clinically diagnosed by experienced doctors according to International Classification of Diseases (ICD)-10 criteria and recruited from Sumiyoshi Hospital (Koufu-shi) and the Nanko Clinic of Psychiatry (Shirakawa-shi), both located in Japan; the mean ± SD age of patients was 28.2±4.4 years. All were medicated outpatients, treated with olanzapine (60%; *n* = 15), aripiprazole (40%, *n* = 10), risperidone long acting injection (16%, *n* = 4), levomepromazine (8%; *n* = 2), blonanserin (8%; *n* = 2), paliperidone (8%; *n* = 2), quetiapine (4%; *n* = 1), fluphenazine (4%; *n* = 1), risperidone (4%; *n* = 1), or sulpiride (4%; *n* = 1). Eleven patients (44%) had taken more than 2 kinds of antipsychotics. Age-matched healthy human volunteers (*n* = 27), including 12 men and 15 women with a mean ± SD age of 26.5±5.6 years, comprised the control group.

**Table 1:** Characteristics of subjects.

	Controls		Patients		*p* value
Number of subjects	27		25		
Gender (male/female)	12/15		11/14		0.8065
Age (year)	26.5±5.6	(18–37)	28.2±4.4	(21–35)	0.251
Onset age (year)			21.4±4.9	(15–30)	
Duration of illness (month)			84.5±57.8	(1–240)	
Chlorpromazine equivalent (mg)			605±345	(150–1455)	
Diabetes mellitus (Y/N)	0/27		0/25		
Smoker/nonsmoker	1/26		10/15		<0.001
BMI (kg/m^2^)	21.4±2.8	(17.4–27.8)	24±4.1	(14.4–31.2)	0.014

The comparison between 2 groups was performed using the χ^2^ test for gender difference and smoking, and the Student’s *t*-test for age and body mass index (BMI).

Ethics statement: Written informed consent was obtained from all subjects prior to participation, and the protocol was approved by the ethics committee in the Faculty of Pharmaceutical Sciences, Toho University (approval number 23-1, 24-6). The capacities of all patients to provide consent were established by psychiatrists with more than 10 years’ experience. In this study, there were no surrogate consent procedures required.

### 2. Blood sampling

Five milliliters of blood was drawn from the arm vein of participants at 11∶00 AM–12∶00 PM before lunch to avoid the effect of food intake wherever possible, and collected in Venoject-II AUTOSEP tubes (Terumo Corporation; Tokyo, Japan); samples were allowed to stand for 30 min at room temperature, consistent with a previous procedure for serum D-serine analysis [6], [10]. Tubes were then centrifuged at 1200×*g* for 15 min. The serum obtained was divided into aliquots of 100 µL each in screw-capped vials, which were stored at –80°C.

### 3. Determination of targeted metabolites in serum

A total of 34 target metabolites were examined: L-tryptophan (Trp), L-kynurenine (Kyn), kynurenic acid (KYNA), 5-hydroxytryptamine (5-HT), D-serine (D-Ser), D-lactate, L-lactate, 3-hydroxybutyrate (3-HB), GSH, γ-glutamylcysteine (GluCys), cysteine (Cys), cysteinylglycine (CysGly), L-amino acids (His, Arg, Gln, Ser, Asn, Glu, Gly, Pro, Thr, Ala, Leu, Ile, Val, Phe, Lys, and Tyr), fatty acids [linoleic acid (LA), arachidonic acid (AA), oleic acid, linolenic acid, and palmitoleic acid], and glucose. The serum levels of these metabolites were determined by using high-performance liquid chromatography (HPLC) methods. An HPLC with fluorescence detection method was used for quantification of L-amino acids [11]–[13], thiol compounds [14], D- and L-Ser [6], [15], D- and L-lactate [16], 3-HB [17], [18], fatty acids [19], and KYNA [20], [21].

Serum Trp and Kyn levels were determined using an HPLC with mass spectrometric detection method as described previously [22], and 5-HT levels were determined using HPLC-electrochemical detection [23]. Glucose levels were determined using a commercial kit (Wako; Osaka, Japan). These methods have already been validated at the serum level for each metabolite.

### 4. Statistical analyses

The non-parametric Mann-Whitney U-test (2-tailed) was performed for statistical analysis of metabolite concentrations, with *p*<0.05 considered significant. The Spearman rank correlation test was performed to determine statistical correlations between variables.

For multivariate analysis, the collected data were imported to SIMCA 13.0.3 software (Umetrics; Umea, Sweden), and an orthogonal partial least square-discrimination analysis (OPLS-DA) was carried out. The statistics, *R* ^2^ (cumulative) and *Q* ^2^ (cumulative), were calculated by OPLS-DA. *R* ^2^ and *Q* ^2^ represent explanatory variable estimating the goodness of fit of the model and predictor, which is the 7-fold cross-validated predictive ability, respectively. The statics range (minimum–maximum) is 0–1. Receiver operating characteristics (ROC) curve tests for metabolites were performed using GraphPad Prism 6 (GraphPad Software, Inc.; La Jolla, CA, USA).

## Results

### 1. Subject characteristics

For the purpose of early detection of schizophrenia, young patients, aged 21–35 years, were enrolled in the present study. In addition, age- and gender-matched healthy subjects were enrolled as controls. As shown in Table 1, no significant difference in sex ratio or age was observed between controls and patients (*p* = 0.807 and *p* = 0.251, respectively).

### 2. Discrimination of controls and patients

**Figure 1:**
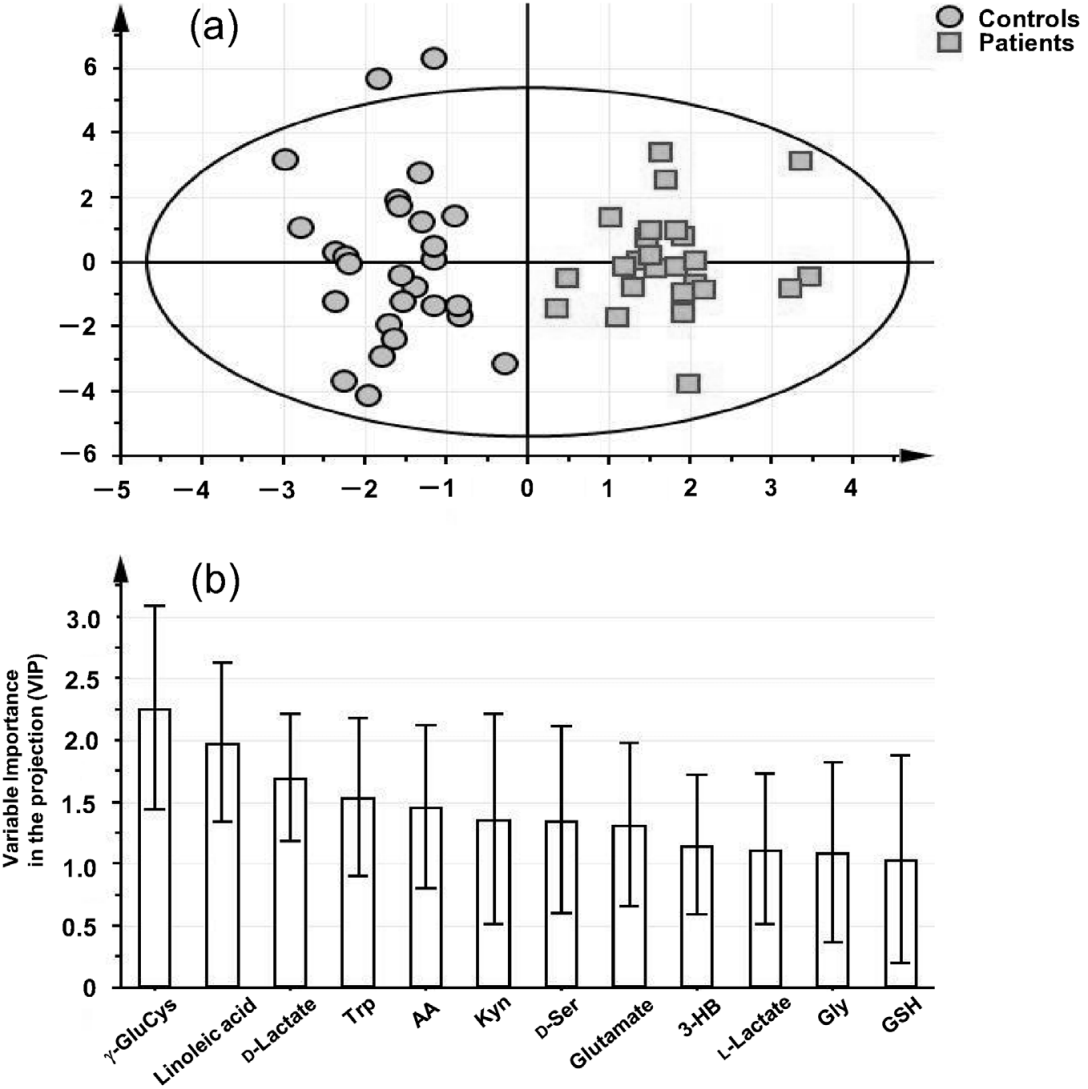
Score plot derived from orthogonal partial least square-discriminant analysis (OPLS-DA) based on the serum levels of 34 targeted metabolites in the subjects (a), and variable importance in the projection (VIP) data of the compounds with VIP values above 1.0 (b).

A total of 34 serum metabolites were quantified from controls and from schizophrenia patients. A multivariate analysis, OPLS-DA, was then carried out, and the results are shown in Fig. 1a. Clear separation between controls and patients was achieved with an *R^2^* (cumulative) and a *Q^2^* (cumulative) of 0.8606 and 0.7203, respectively. For *Q^2^*, a predictor calculated by cross-validation, a value above 0.4 indicates that the OPLS-DA model is reliable; these results showed that patients could be discriminated from controls using these serum metabolite levels. Among the 34 compounds tested, 12 had a variable importance in the projection (VIP; an indicator of discriminatory power) value above 1.0; these were γ-GluCys, LA, D-lactate, Trp, AA, Kyn, D-Ser, Glu, 3-HB, L-lactate, Gly, and GSH (Fig. 1b). The relatively large VIP values of γ-GluCys (2.26), LA (1.98), and D-lactate (1.70) showed that these metabolites were the main contributors to the patient-group separation in this study.

### 3. Comparison of serum levels between controls and patients

The serum level results are categorized into 5 separate tables (Tables S1, S2, S3, S4, and S5). The metabolites for which serum levels differed between controls and schizophrenia patients are listed in Table 2. With regard to serum amino acids, the levels of Glu (*p* = 0.0145), D-Ser (*p* = 0.00163), Tyr (*p* = 0.0469), and Thr (*p* = 0.0286) were significantly altered between patients and controls. Serum levels of D-lactate (*p* = 2.43×10^−5^) and 3-HB (*p* = 0.00438) were significantly different between the patient and control groups. The analysis of thiol compounds and fatty acids, respectively, revealed that levels of GSH (*p* = 0.0344), γ-GluCys (*p* = 1.75×10^−6^), LA (*p* = 1.61×10^−5^), and AA (*p* = 0.00149) were significantly altered in patients. With regard to Trp metabolites, serum levels of Trp (*p* = 0.00101), Kyn (*p* = 0.00568), and 5-HT (*p* = 0.0131), but not KYNA (*p* = 0.6145), were significantly altered.

**Table 2:** Serum levels of metabolites (µmol/L) that were differentially expressed between controls and schizophrenia patients.

Compound	Levels in serum		
	Controls (*n* = 27)	Schizophrenia patients (*n* = 25)	*p* value*
γ–Glutamylcysteine	3.05±0.11	2.07±0.12	1.746E-06^a^
Linoleic acid	264±17.0	159±12.0	1.610E-05^a^
D-Lactate	7.26±0.481	13.1±1.42	2.426E-05^a^
L-Tryptophan	81.3±3.36	101±4.48	0.001011^a^
Arachidonic acid	55.4±4.48	38.5±2.18	0.001485
L-Kynurenine	1.74±0.121	2.35±0.162	0.005676
D-Serine	1.88±0.07	1.57±0.07	0.00163
Glutamate	35.5±3.20	71.3±12.3	0.0145
3-Hydroxybutyrate	46.5±10.1	17.6±3.84	0.004378
Glutathione	3.67±0.241	3.03±0.149	0.03437
5-Hydroxytryptamine	0.85±0.077	0.61±0.096	0.01308
Threonine	126±7.44	111±10.3	0.0286
Tyrosine	65.1±2.55	57.2±3.33	0.0469

Only *p* values below 0.05 are included in this table.

*Non-parametric Mann-Whitney *U*-test.

a Significantly different following Bonferroni correction (<0.05/34).

In summary, the serum levels of 9 compounds, γ-GlyCys, LA, AA, D-Ser, 3-HB, GSH, 5-HT, Thr, and Tyr, were significantly decreased, whereas those of 4 compounds, D-lactate, Trp, Kyn, and Glu, were significantly increased in patients compared to controls.

When a Bonferroni correction was applied, levels of 4 metabolites (γ-GluCys, LA, D-lactate, and Trp) were still significantly different between control and patient groups, while the following metabolites were present at similar levels in both groups: AA, Kyn, D-Ser, Glu, 3-HB, GSH, 5-HT, Thr, and Tyr (Table 2).

Similar to a previous study [10], there was a higher number of smokers among enrolled schizophrenia patients than controls (Table 1). There were no significant differences in the serum levels of metabolites between smokers and non-smokers with the exception of γ-GluCys (*p* = 0.035) (Table S6). Regarding γ-GluCys, however, significant differences between control and patient groups were observed in both non-smoking (*p* = 5.71E-07) and smoking patients (*p* = 0.0152, Bonferroni test).

### 4. ROC curve analysis

**Figure 2:**
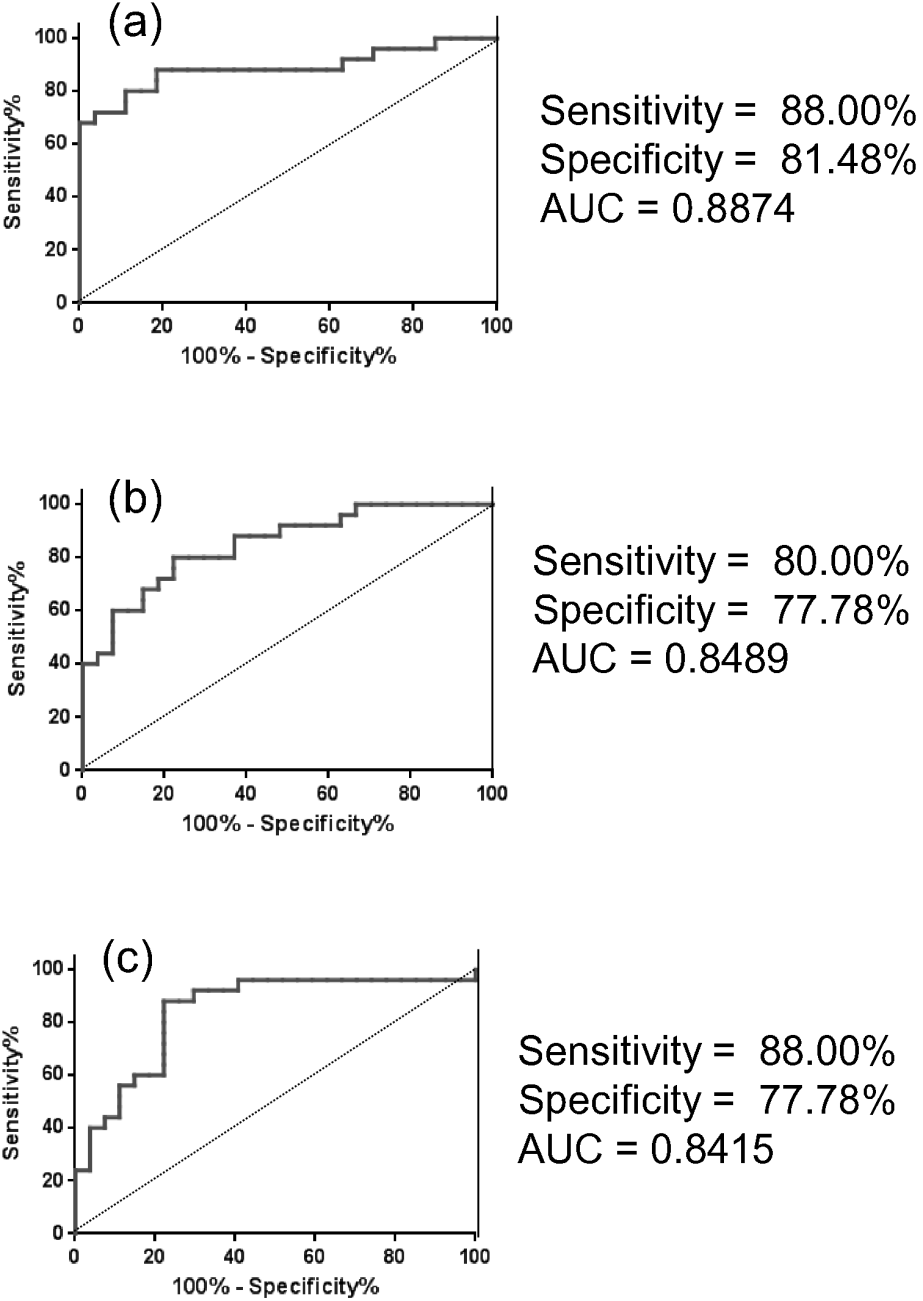
Receiver operating characteristic (ROC) curve analyses of γ-GluCys (a), lenoleic acid (b), and D-lactate (c).

The ROC curves of serum levels of γ-GluCys, LA, and D-lactate, which displayed relatively large VIP values, are shown in Fig. 2. The AUC values for these metabolites were 0.8874, 0.8489, and 0.8415, respectively. The sensitivity and specificity at the cut-off points for γ-GluCys, LA, and D-lactate were 88.00 and 81.48%, 80.00 and 77.78%, and 88.00 and 77.78%, respectively.

### 5. Correlations of the serum levels in controls and patients

**Figure 3:**
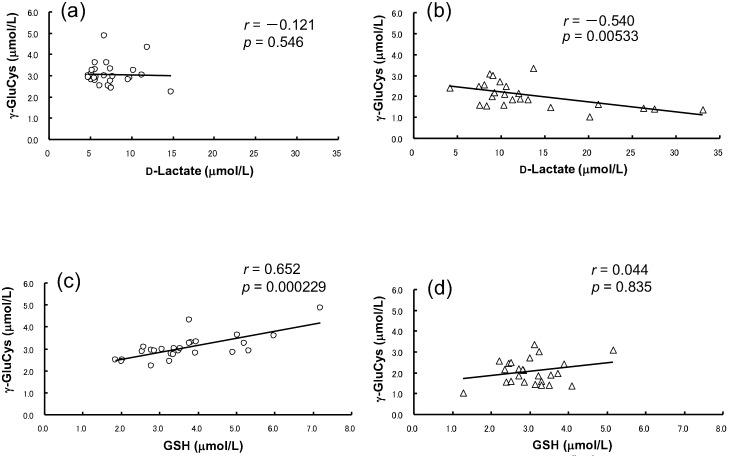
Correlation plots of D-lactate and γ-GluCys serum levels in controls (a) and in patients with schizophrenia (b), and between GSH and γ-GluCys in controls (c) and in patients with schizophrenia (d).

Among the 13 compounds that discriminated the control and patient groups (Table 2), a negative correlation between D-lactate and γ-GluCys levels was observed (*p* = 0.00533, *r* = –0.5400) in patients (Fig. 3b), but not in controls (Fig. 3a). A positive correlation between γ-GluCys and GSH was observed in controls (*r* = 0.652, *p* = 0.000229) (Fig. 3c), but not in patients (*r* = 0.044, *p* = 0.8351) (Fig. 3d).

Serum Tyr and 5-HT levels in patients were significantly correlated with daily chlorpromazine (CP) equivalents, and the levels of D-Ser, Glu, 3-HB, AA, and LA were positively correlated with duration of illness (Table 3).

**Table 3:** Correlations between serum levels of metabolites and CP equivalent (mg) or duration of illness (months).

	Tyr	5-HT			
CP equivalent	*r* = –0.488	*r* = 0.397			
(mg)	(*p* = 0.0133)	(*p* = 0.0491)			
	D-Ser	Glu	3-HB	Arachidonic acid	Linoleic acid
Duration of illness	*r* = 0.478	*r* = 0.397	*r* = 0.540	*r* = 0.630	*r* = 0.415
(months)	(*p* = 0.0157)	(*p* = 0.0491)	(*p* = 0.0064)	(*p* = 0.0007)	(*p* = 0.0390)

## Discussion

Our data revealed that analysis of 34 serum metabolites was sufficient to discriminate between controls and schizophrenia patients by OPLS-DA (Fig. 1 (a)), and that the decreased levels of γ-GluCys and increased levels of D-lactate were the major contributors to this discrimination (Fig. 1 (b)). To the best of our knowledge, there have been no reports to date showing that serum levels of γ-GluCys or D-lactate are altered in schizophrenia patients. By analyses of ROC curves (Fig. 2), the serum levels of these metabolites revealed sufficient AUC values (0.8874 and 0.8415) to discriminate patients from controls. The sensitivity and selectivity values were also satisfactory (Fig. 2).

Oxidative stress may be involved in the pathogenesis of schizophrenia [8], [24]. On the other hand, carbonyl stress, such as the glycation and inactivation of proteins by the dicarbonyl compound methylglyoxal (MGO), is involved in diabetic complications [25], [26]. In the glyoxalase pathway, glyoxalase I converts MGO to *S*-lactoylglutathione, and subsequently, *S*-lactoylglutathione is converted to D-lactate by glyoxalase II [25] (Fig. S1). Increased d-lactate levels have been found in diabetes mellitus, likely due to enhanced MGO production [25]. Indeed, we previously reported a significant increase in the serum d-lactate level in diabetic patients [16]. Recently, it was reported that carbonyl stress was enhanced in a subpopulation of schizophrenia patients due to a decline in glyoxalase I activity [27]. Therefore, a reduction in glyoxalase I activity would be expected to lead to a lower serum D-lactate level. However, our current data show that the serum D-lactate level was significantly increased in patients. Although further experiments are required, we suggest that increased activity of glyoxalase II in patients may underlie the increased D-lactate level.

Another finding in the present study is that the serum γ-GluCys level was remarkably reduced in schizophrenia patients. γ-GluCys is a precursor of an endogenous antioxidant, GSH, and is produced from Glu and Cys by glutamatecysteine ligase (GCL; Fig. S2). GCL activity is a rate-limiting enzyme for GSH synthesis, and genetic polymorphisms in GCL significantly modulate schizophrenia risk [28], [29]. As shown in Table 2, the low GSH level observed in patients is consistent with previous reports [9], [30]. However, to the best of our knowledge, we are the first to report a decreased level of the GSH precursor γ-GluCys in the sera of schizophrenia patients. In addition, the level of Glu, which is a precursor of γ-GluCys, was significantly increased in patients (Table 2), suggesting that GCL activity might also be attenuated. The significant decrease in the GSH level of patients indicates that anti-oxidant defenses against reactive oxygen species (ROS) and reactive nitrogen species (RNS) may be compromised in schizophrenia. Interestingly, a significant negative correlation between D-lactate and γ-GluCys was observed specifically in the patients’ sera (Fig. 3b). Since *S*-lactoylglutathione is metabolized by glyoxalase II to release D-lactate and GSH (Fig. S2), elevated GSH levels would be expected in patient serum; however, we observed low serum GSH levels in patients (Table 2). In addition, a positive correlation between serum γ-GluCys and GSH levels was observed specifically in controls (Fig. 3c and d). Taken together, these data suggest that reduced GSH levels in schizophrenia patients may be due to the rapid consumption of GSH during its anti-ROS or -RNS activities.

Recently, an increased 3-HB level was observed in serum from schizophrenia patients in a metabonomics study [2]. Conversely, Cai et al. (2012) reported a decreased 3-HB level in the serum of patients. As shown in Table 2, we found that 3-HB levels were reduced in patient serum. As a ketone body, 3-HB is produced from acetoacetate, which in turn is derived from fatty acids (Fig. S3). 3-HB levels increase as a result of fasting and in diabetic subjects due to limited utilization of glucose; under these conditions, there is instead enhanced utilization of ketone bodies for energy production. A recent report showed that 3-HB-dependent increases in transcription of the oxidative stress resistance factors FOXO3A and MT2 constitute an anti-oxidant pathway [31]. Thus, depletion of endogenous 3-HB in patients may induce oxidative stress, which is implicated in schizophrenia etiology [8].

Regarding PUFAs, we found that serum AA and LA levels were significantly decreased in patients (Table 2). These results are consistent with previous reports of low PUFA levels in schizophrenia patients [32]. LA is initially obtained from the daily diet, and is then converted to AA by elongase and desaturase (Fig. S3). Considering these facts, it is likely that the reduction in PUFA levels in schizophrenia patients is caused by their excess oxidative stress. In addition, it has been reported that increased tissue expression of fatty acid binding protein (FABP) is closely associated with the etiology of schizophrenia [33], and both LA and AA are bound by FABP7 [33], [34]. Therefore, FABP7-dependent sequestering of circulating PUFAs in biological fluids might also contribute to reduced serum PUFA levels in patients.

In the present study, we examined Trp and its metabolites, Kyn, KYNA, and 5-HT, since the associated metabolic pathways produce several neuroactive substances [35], [36]. Our data revealed that serum Trp and Kyn levels were significantly increased in patients (Table 2). Trp is metabolized to Kyn by tryptophan 2,3-dioxygenase or indoleamine 2,3-dioxygenase in the Kyn pathway, and to 5-HT by tryptophan hydrolase (TH) and aromatic amino acid decarboxylase (AAD) in the serotonin pathway (Fig. S4) [37], [38]. Since only medicated patients were enrolled in the present study, our present results regarding serum Trp levels are consistent with the report of Xuan et al. [4], who found increased Trp levels in the serum of medicated patients. By contrast, the 5-HT level was significantly decreased in patients compared to controls (Table 2). Therefore, activities of TH or AAD might also be decreased in patients. 5-HT is further metabolized to melatonin, which possesses anti-oxidant activity [36], [39]. We speculate that the decreased level of 5-HT will lead to lower melatonin levels, and thus compromised antioxidant defenses, in patients.

Conversely, the serum KYNA level was not altered significantly between controls and patients (*p* = 0.6145), although increased KYNA levels in the post-mortem brain tissue [40] or cerebrospinal fluids [41] from schizophrenia patients have been reported. KYNA acts as an endogenous antagonist of the glycine site of the NMDA receptor. However, we found no alteration in the serum KYNA levels of patients, indicating that increased KYNA levels might occur only in the CNS. By contrast, we did observe a significant decrease in serum levels of D-serine, a co-agonist of the glycine site of the NMDA receptor [5], [42] (Table 2), which is consistent with previous reports [6], [10]. Genetic studies of schizophrenia show that the susceptibility gene G72 plays a role in regulation of D-amino acid oxidase (DAO), an enzyme that is upregulated in schizophrenia [43], [44]. Thus, high-level expression of DAO in several tissues, including the brain, liver, and kidney, may underlie the observed decreased serum D-serine level in patients.

In this study, we were only able to include medicated patients, as recruitment of drug-naïve patients was not possible. Therefore, we cannot exclude the potential effects of medication in the interpretation of our results. Serum Tyr and 5-HT levels were significantly correlated with daily CP, and the levels of both metabolites were significantly lower in patients. The negative correlation between the serum Tyr level and CP equivalent is likely due to a medication-induced effect. Tyr is produced from Phe by phenylalanine hydroxylase (PH), and is then converted to catecholamines (e.g., dopamine and noradrenaline) by several enzymes such as tyrosine hydrolase and dopa decarboxylase. Since the serum level of Phe was not altered (Table S1), the involvement of decreased activity of PH or enhanced enzyme activities of catecholamines biosynthesis might occur in patients.

By contrast, the serum 5-HT level was positively correlated with CP equivalents. 5-HT is sensitive to oxidation; thus, elevated ROS or RNS in patients could lead to non-enzymatic decomposition of 5-HT in vivo. The second generation antipsychotics (SGA) aripiprazole and quetiapine have indirect anti-oxidative effects via induction of the anti-oxidative enzyme, superoxide dismutase [45]. Thus, the positive correlation between 5-HT levels and CP equivalents may be due to the SGA-dependent suppression of 5-HT oxidation.

The serum levels of D-Ser, Glu, 3-HB, AA, and LA exhibited a significant positive correlation with duration of illness (Table 3). Since the serum Glu level is reportedly increased even in drug-naïve patients [46], the effect of medication on the Glu level might be negligible. Calcia et al. [10] reported that plasma D-Ser levels were lower in non-medicated than in medicated patients. Consistent with this, D-Ser levels were higher in patients with a longer history of recorded illness, suggesting that sustained medication use may increase D-Ser levels. Indeed, we observed that SGAs possess DAO-inhibitory activity in vitro (data not shown). Furthermore, the anti-oxidant effects of SGAs [45] may explain why prolonged treatment of patients restores the levels of 3-HB, AA, and LA, as each of these metabolites are sensitive to oxidative stress.

There are nonetheless limitations to the current study. In the present study, blood samples were not collected from subjects in the early morning in a fasting state. Therefore, future studies should be performed under this condition to exclude the possibility that feeding is a confounding variable. Next, we only studied relatively young patients (≤35 years old), and we therefore cannot extrapolate our findings to a broader patient range. In addition, all patients were medicated, and it is therefore important to gather information from drug-naïve patients in future studies.

## Conclusion

The present finding that the serum levels of 13 metabolites may be differentially regulated in schizophrenia patients extends our knowledge of the pathophysiology of the disease. Building upon this study, future investigations could identify which metabolites are suitable biomarkers for the early detection and prognosis of schizophrenia and its associated treatment regimens.

## Supporting Information

Figure S1 **D-Lactate-associated biosynthetic and metabolic pathways.** Rectangles denote the quantified compound, and bold red rectangles denote metabolites that were differentially altered in patients compared to controls. Red upward or downward arrows indicate that the level increased or decreased, respectively.
(TIF)

Figure S2 **Relevant biosynthetic and metabolic pathways associated with GSH.** Rectangles denote quantified compounds, and bold red rectangles denote the metabolites that were differentially altered in the serum of patients versus controls. Red upward or downward arrows indicate that the level increased or decreased, respectively.
(TIF)

Figure S3 **Relevant biosynthetic and metabolic pathway of PUFAs.** Rectangles denotes the compounds that were quantified, and bold red rectangles denote the metabolites that differed between patients and controls. Red downward arrow means that the level decreased.
(TIF)

Figure S4 **Relevant metabolic pathway of Trp.** Rectangle denotes compounds quantified and bold red rectangle denotes the compounds whose levels were altered in patients compared to healthy controls. Red upward or downward arrows indicate that the level increased or decreased, respectively.
(TIF)

Table S1 **Serum levels of amino acids (including D-serine) in controls and patients with schizophrenia.**
(XLS)

Table S2 **Serum levels of glucose, D-lactate, L-lactate, and 3-hydroxybutyrate (3-HB) in controls and patients with schizophrenia.**
(XLS)

Table S3 **Serum levels of thiol compounds in controls and patients with schizophrenia.**
(XLS)

Table S4 **Serum levels of unsaturated fatty acids in controls and patients with schizophrenia.**
(XLS)

Table S5 **Serum levels of Trp and its metabolites in controls and patients with schizophrenia.**
(XLS)

Table S6 **Serum levels of metabolites (µmol/L) in smoking and non-smoking schizophrenia patients.**
(XLS)
