# Supplementary figures and images for: Quantitative Analyses of Schizophrenia-Associated Metabolites in Serum: Serum D-Lactate Levels Are Negatively Correlated with Gamma-Glutamylcysteine in Medicated Schizophrenia Patients
Source: PLoS One. 2014 Jul 8;9(7):e101652. doi: 10.1371/journal.pone.0101652 (PMC4086900; doi:10.1371/journal.pone.0101652)

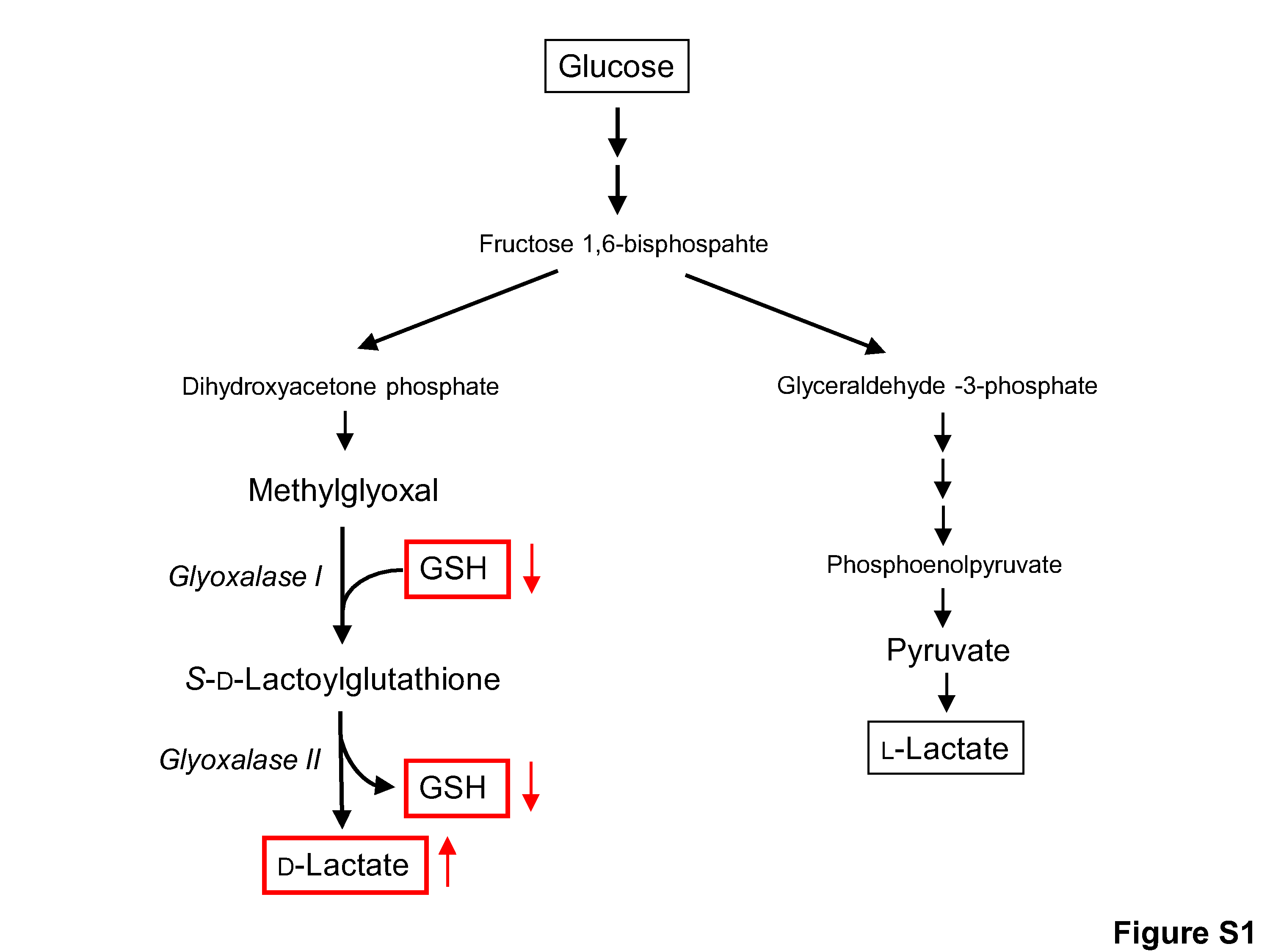

Supplement: Figure S1 — D-Lactate-associated biosynthetic and metabolic pathways. Rectangles denote the quantified compound, and bold red rectangles denote metabolites that were differentially altered in patients compared to controls. Red upward or downward arrows indicate that the level increased or decreased, respectively. (TIF) [file pone.0101652.s001.tif]

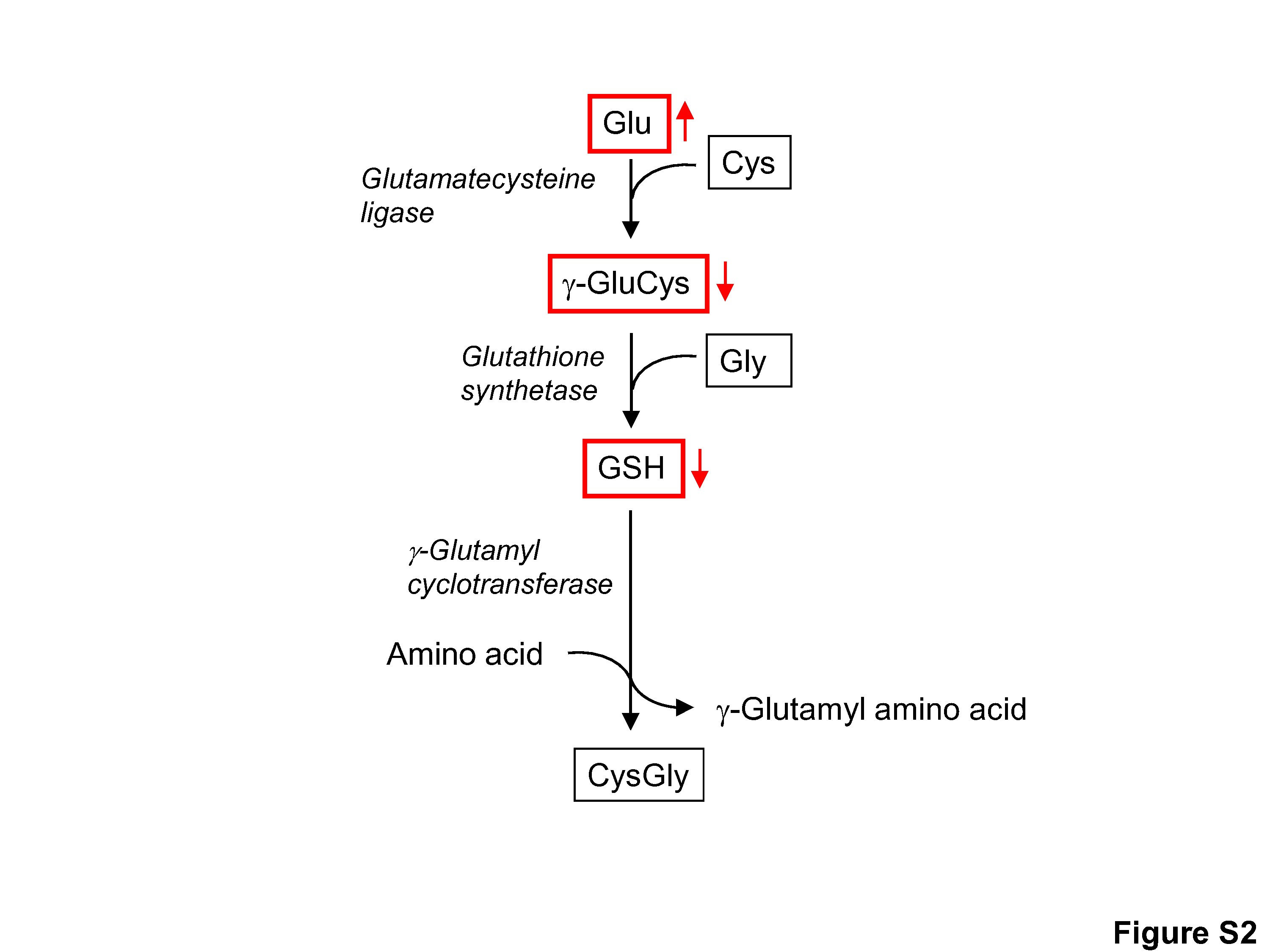

Supplement: Figure S2 — Relevant biosynthetic and metabolic pathways associated with GSH. Rectangles denote quantified compounds, and bold red rectangles denote the metabolites that were differentially altered in the serum of patients versus controls. Red upward or downward arrows indicate that the level increased or decreased, respectively. (TIF) [file pone.0101652.s002.tif]

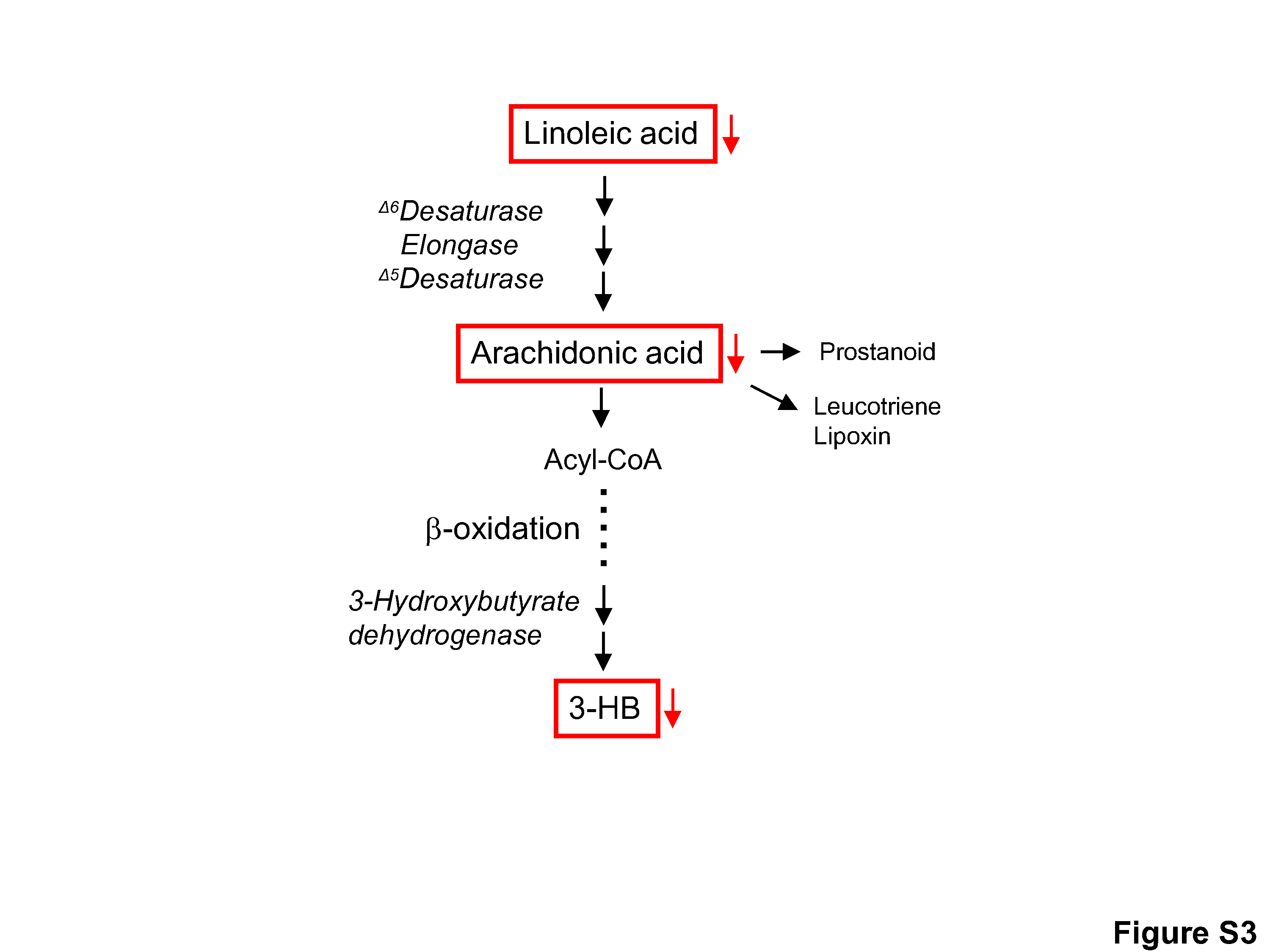

Supplement: Figure S3 — Relevant biosynthetic and metabolic pathway of PUFAs. Rectangles denotes the compounds that were quantified, and bold red rectangles denote the metabolites that differed between patients and controls. Red downward arrow means that the level decreased. (TIF) [file pone.0101652.s003.tif]

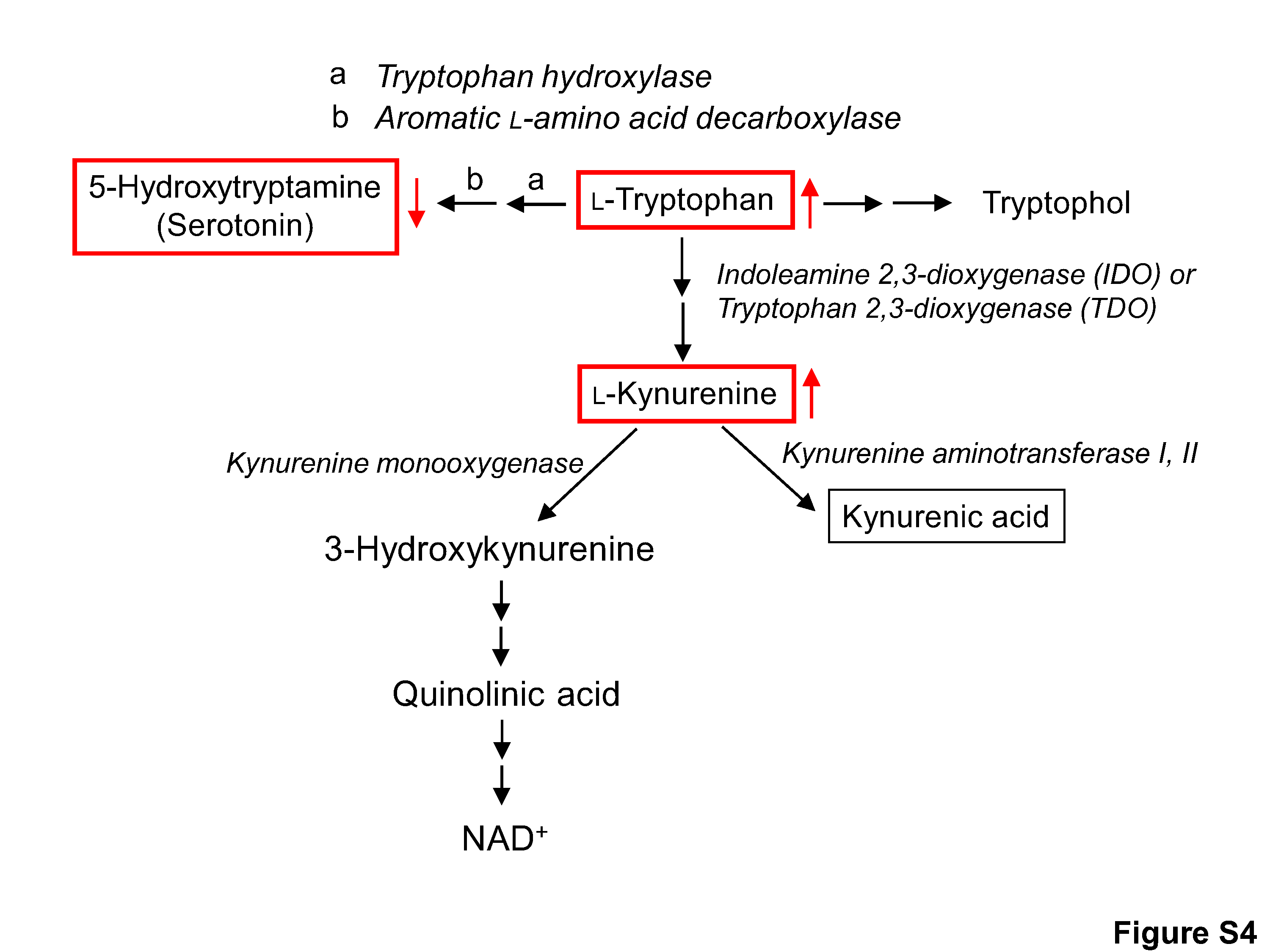

Supplement: Figure S4 — Relevant metabolic pathway of Trp. Rectangle denotes compounds quantified and bold red rectangle denotes the compounds whose levels were altered in patients compared to healthy controls. Red upward or downward arrows indicate that the level increased or decreased, respectively. (TIF) [file pone.0101652.s004.tif]
